# PReS-FINAL-2040: Outcome of macrophage activation syndrom (MAS) in systemic juvenile idiopatic arthritis (SJIA) in non biologic treated patient

**DOI:** 10.1186/1546-0096-11-S2-P53

**Published:** 2013-12-05

**Authors:** DS Lazarevic, J Vojinovic

**Affiliations:** 1 Pediatric Rheumatology, Clinic of Pediatrics, Clinical Center Nis, Nis, Serbia

## Introduction

MAS is serious and severe, sometimes life threatening complication of sJIA but data about survivor outcomes are missing.

## Objectives

We present 16 year old girl who developed MAS six years after sJIA was established and whose parents refused some treatment options.

## Methods

Analiysis of clinical outcome and laboratory parametars in non biologic treated patient with sJIA who developed MAS.

## Results

After episode of fever and exudative pericarditis (cardiac tamponade) in 2^nd ^and 4^th ^year of life the diagnose of sJIA was established and steroid and NSAIDs therapy was introduced. Clinical remission was achieved and lasted 3 years when at the age of years, after moderate respiratory infection she developed persistant oligoarthritis why metotrexate was administrated during one year until disease remission. She was lost for follow up until admitted to the hospital due to high grade fever, macular rash, weakness, oligoarthritis, aphthous stomatitis, cervical limfadenopathy and pericarditis. The pulses of methyl-prednisolone were started followed with the oral steroids (2 mg/kg) and NSAIDs therapy. Episodes of fever, rash and morning stiffness with elevated values of ESR, CRP and WBC were still present after 2 weeks why CyA was added. Next 3 weeks she was relatively stabile, but became Coushing-oid with occasional fever and high blood pressure, In the fourth week of hospitalization she developed seizures due to hypertensive encephalopathy (TA 210/160 mmHg). Intensive antihypertensive and antiedematous therapy has normalised blood pressure without new episodes of seizures. After 6 weeks of treatment she developed intensive epigastric pain and diffuse tenderness in the abdomen (peritonitis) with diffuse purpuric skin lesions all over the body. Laboratory results have shown low PLT count, hypertriglyceridiemia highly elevated liver enzymes, feritin and LDH (10403 mmol/L) and profound hyponatriemia (112 mmol/l). Diagnose of MAS was established and due to oliguria and her serous condition hemodiafiltration was initiated together with etoposide and VP16. During next 2 months she was in ICU and have had imapared coagulation parameters and developed necrotic-vasculitis skin changes. Parents refused implementation of any additional immunosuppresive or biologic therapy except shourt course of Thalidomide. Due to a chronic renal failure she is still on regular hemodialysis. During follow up period she never developed new epizodes of arthritis but have developed amyloidosis (with constrictive pericarditis and cardiomyopathy), chronic renal failure and seizures.

## Conclusion

MAS is major cause of mortality in patients with sJIA. Data about MAS survivors and their outcome in correlation to treatment aproach are missing especially for patients not treated with biologics.

## Disclosure of interest

None declared.

